# Influencia del gradiente social sobre la salud bucal de mujeres trabajadoras formales

**DOI:** 10.15649/cuidarte.2334

**Published:** 2023-03-29

**Authors:** Andrea Johanna Almario-Barrera, Sonia Constanza Concha-Sánchez

**Affiliations:** 1 . Universidad Santo Tomás seccional Bucaramanga, Colombia. E-mail: almita 1613@hotmail.com Universidad Santo Tomás Universidad Santo Tomás Colombia almita 1613@hotmail.com; 2 . Universidad Santo Tomás seccional Bucaramanga, Colombia. E-mail: sococosa@yahoo.com Universidad Santo Tomás Universidad Santo Tomás Colombia sococosa@yahoo.com

**Keywords:** Gradiente Social, Posición Social, Mujer, Salud Bucal, Trabajo Formal., Social Gradient, Social Position, Woman, Oral Health, Formal Work., Saúde Bucal, Gradiente Social, Mulheres, Posigao Social, Trabalho Formal

## Abstract

**Introducción:**

Las patologías bucales se asocian con las condiciones sociales, materiales y el nivel socioeconómico desfavorables. La salud bucal de las mujeres se ve afectada por aspectos sociales, que marcan inequidades en salud, si se compara con los hombres.

**Objetivo:**

Evaluar la influencia del gradiente social sobre la salud bucal de mujeres trabajadoras de una universidad de Santander, Colombia.

**Materiales y Métodos:**

Estudio observacional analítico de corte transversal que involucró a 84 mujeres trabajadoras. Se utilizaron variables sociodemográficas, de condición de salud bucal (presencia de caries dental, enfermedad periodontal y edentulismo) y posición social. Se estableció una relación entre estas. Para ello, se aplicaron las pruebas Chi cuadrado o Exacto de Fisher, t de Student o test de rangos de Wilcoxon, con una significancia a<0,05.

**Resultados:**

Se evidenció una prevalencia de caries dental de un 85,7%, enfermedad periodontal de un 79,8%, y prevalencia de edentulismo de un 40,5%; los factores sociales que se presentaron con mayor frecuencia mostrando alguna relación con las condiciones bucales fueron edad, etnia, estado civil, nivel educativo, la labor que realiza diariamente, estrato socioeconómico, la responsabilidad económica dentro del hogar.

**Conclusión:**

El gradiente social no registró diferencias estadísticamente significativas al analizarlo con las patologías orales; sin embargo, se estableció que las mujeres que se ubicaron en la posición social alta tenían menos carga de enfermedades bucales; mientras que las mujeres que se encontraban en la posición social baja tenían mayor prevalencia de caries dental, enfermedad periodontal y edentulismo

## Abstract (en)

**Introduction:**

Oral diseases are associated with unfavorable social, material, and socioeconomic conditions. Women's oral health is affected by social aspects, which increase health inequalities when compared to men.

**Objective:**

To evaluate the social gradient's influence on the oral health of working women at a university in Santander, Colombia.

**Materials and methods:**

Analytical cross-sectional observational cross-sectional study involving 84 working women. Sociodemographic variables, oral health condition variables (presence of dental caries, periodontal disease, and edentulism), and social status variables were used to establish relationships. Chi-square test or Fisher's Exact test, Student's t-test or Wilcoxon rank test were used, with a significance a<0.05.

**Results:**

The prevalence of dental caries was 85.7%, periodontal disease 79.8%, and edentulism 40.5%. Age, ethnicity, marital status, educational level, work carried out daily, socioeconomic status, and responsibility for household finances were the social factors most frequently related to oral conditions.

**Conclusion:**

The social gradient did not register statistically significant differences when analyzed with oral diseases. However, it was established that women with a high social status had a lower burden of oral diseases, while women with a low social status had a higher prevalence of dental caries, periodontal disease, and edentulism.

## Resumo

**Introdugáo:**

As patologías bucais estao associadas a condigóes sociais, materiais e socioeconómicas desfavoráveis. A saúde bucal das mulheres é afetada por aspectos sociais, que marcam as iniquidades em saúde, quando comparadas aos homens.

**Objetivo:**

Avaliar a influencia do gradiente social na saúde bucal de mulheres trabalhadoras de uma universidade em Santander, Colómbia.

**Materiais e métodos:**

Estudo observacional analítico transversal envolvendo 84 mulheres trabalhadoras. Foram utilizadas variáveis sociodemográficas, condigao de saúde bucal (presenga de cárie dentária, doenga periodontal e edentulismo) e posigao social. Estabeleceu-se uma relagao entre eles. Para isso, foram aplicados os testes Qui-quadrado ou Exato de Fisher, Teste T de Student ou teste de faixas de Wilcoxon, com significancia de a<0,05.

**Resultados:**

Evidenciou-se prevalencia de cárie dentária de 85,7%, doenga periodontal de 79,8% e prevalencia de edentulismo de 40,5%; os fatores sociais que apareceram com maior frequencia, mostrando alguma relagao com as condigóes bucais, foram idade, etnia, estado civil, escolaridade, trabalho diário, nível socioeconómico, responsabilidade económica dentro do lar.

**Conclusao:**

O gradiente social nao registrou diferengas estatisticamente significativas ao analisá-lo com patologias bucais; no entanto, verificou-se que as mulheres que se encontravam em posigao social elevada apresentavam menor carga de doengas bucais; enquanto as mulheres que estavam em posigao social baixa apresentaram maior prevalencia de cárie dentária, doenga periodontal e edentulismo.

## Introducción

En la actualidad las mujeres trabajadoras, constituyen la mitad de la fuerza laboral en el mundo, sin embargo, las condiciones de trabajo, así como su acceso a las oportunidades de progreso difieren de las de los hombres debido a que las mujeres están en desventaja con relación a los hombres en cuanto a los trabajos que les ofrecen y la cuantía de remuneración que reciben; por otra parte, muchas mujeres renuncian o restringen el empleo debido a sus responsabilidades familiares, asociadas a los roles de género que les atribuye la sociedad^1^. La eliminación de los obstáculos y desigualdades que enfrentan las mujeres en el ámbito del trabajo son un avance hacia la realización de su potencial personal, económico y social; así como del desarrollo financiero y cultural de una región y un país. La participación laboral de las mujeres, principalmente de las que no son cabeza de hogar, ha sido un tema muy importante en el campo de la economía laboral debido a su incremento exponencial a nivel mundial.

En Colombia, según los datos reportados por el Departamento Administrativo Nacional de Estadística (DANE) para el primer trimestre de 2009, el 40,8% de la población trabajadora estuvo conformada por mujeres. Sin embargo, para el mismo trimestre de 2016 se observó un aumento de 2,3% comparado con el dato anterior, lo que permite evidenciar que la participación de la mujer registró un incremento en el ámbito laboral^2^. Lo que permite evidenciar aspectos relacionados con la oportunidad de acceso al trabajo y a una remuneración inadecuada, ya que se evidencia un panorama de desigualdades en el desarrollo laboral de las mujeres; y esto genera que en varias ocasiones estas sean injustas, causando que el estado de la salud general y salud bucal, se vean afectados por la injerencia de factores sociales sobre los biológicos y que produzcan inequidades en la salud de las mujeres^3^.

Diferentes problemas sociales afectan la salud de los hombres y las mujeres; y algunas enfermedades muestran un impacto diferencial por género; por lo tanto, es necesario implementar soluciones adecuadas acorde con sus necesidades. Por otra parte, para aquellos trastornos que afectan por igual a ambos sexos, con frecuencia, las mujeres enfrentan mayores dificultades para obtener la asistencia médica u odontológica que necesitan, asociados a las desigualdades de género e inequidades sociales; por ejemplo, en materia de acceso a educación, empleo e ingresos que limitan la capacidad de las mujeres para proteger su propia salud y salud bucal, así como acceder a la atención en salud necesaria para su bienestar^4^.

En lo que respecta a la salud bucal, los estudios han identificado una asociación entre las condiciones sociales desfavorables y problemas orales relacionados con las condiciones materiales de vida y el nivel socioeconómico, tales como insuficiencia de ingresos de la familia, el nivel de educación, la clase social y el grupo étnico^5^ y, por esta razón se relaciona con la realidad política, económica, social y las relaciones de poder dentro de una sociedad^6^^-^^7^.Como se observa en los reportes de los estudios la salud bucal de las mujeres no solo está influenciada por aspectos biológicos pues también se ve afectada por aspectos sociales, que marcan diferencias injustas (inequidades) en salud que son significativas^8^.

Un aspecto importante de inequidad social en salud y en salud bucal que se ha venido estudiando es el gradiente social, el cual se expresa a partir de las deficientes circunstancias sociales y económicas que afectan la salud durante la vida. De acuerdo con este concepto, las personas que están en los estratos sociales más bajos por lo general tienen más riesgo de sufrir enfermedades graves y muerte prematura que quienes están en los estratos sociales altos. La mayoría de las enfermedades y causas de muerte son más comunes en los estratos más bajos de la sociedad. El gradiente social en la salud refleja desventajas materiales y los efectos de la inseguridad, la ansiedad y la falta de integración social. Los modelos de salud muestran un gradiente social continuo, a tal punto que incluso en el personal administrativo subordinado hay más enfermedades y muerte prematura que en el personal de mayor rango^9^^-^^10^, hecho que también se evidencia en el marco de la salud bucal, el cual la compromete, ya que muestra de una manera evidente como la posición social de un individuo tiene una relación lineal inversa entre la posición socioeconómica y la salud, de manera que la distribución desigual de la salud a través de los estratos socioeconómicos se ha observado en los países industrializados y menos desarrollados, y de una manera significativa para las enfermedades más comunes y las causas de muerte^3^.

Por lo cual, al medir el gradiente social en la población se debe tener en cuenta la posición social, el cual, según Arrivillaga “es el lugar simbólico que ocupa una persona en el esquema de la sociedad y que refleja las condiciones del sujeto respecto a los demás integrantes de la comunidad, es decir que es como un grupo de condiciones que actúan conjuntamente para determinar el lugar de una persona en la sociedad”^11^.

Considerando lo expuesto, se plantea la siguiente hipótesis de estudio el gradiente social influye sobre la salud bucal de las mujeres trabajadoras formales de una empresa privada, por lo tanto, el objetivo de este trabajo es evaluar la influencia del gradiente social sobre la salud bucal de mujeres vinculadas laboralmente y relacionar los factores sociales que influyen sobre la salud bucal en este grupo de mujeres.

## Materiales y Métodos

Se realizó un estudio observacional analítico de corte transversal. La población de estudio la constituyeron 581 mujeres trabajadoras pertenecientes a las diferentes dependencias de una universidad privada en Santander y la muestra 84 mujeres vinculadas laboralmente a la institución de estudio. Para el cálculo de la muestra se empleó el paquete estadístico EPIDAT mediante la rutina muestreo en el apartado de cálculo de tamaño de muestra, contraste de hipótesis, comparación de proporciones de grupos independientes, considerando una proporción esperada de caries dental en la población uno (n=117) de servicios generales, secretarias y auxiliares de 84,6% y en la población dos (n=142) docentes y profesionales de apoyo a las diferentes actividades laborales de la universidad de 57,1% con un nivel de confianza del 95% , un efecto de diseño de 1,0 y una potencia de 80%, lo cual se obtuvo a partir de un recalculo de tamaño de muestra donde la población para este recalculo era de 218 mujeres, a partir de la prueba piloto que se realizó con 20 mujeres.

La muestra fue seleccionada mediante un muestreo no probabilístico por conveniencia, ya que resultaba más ajustado a la realidad de las mujeres, examinar y realizar la encuesta a las que tuvieran disponibilidad en el tiempo de aplicación de la investigación y proximidad geográfica al lugar en el que se hacían los exámenes bucales. Se aplicaron criterios de exclusión con gestantes y mujeres que padezcan alguna patología de origen coronario y con antecedentes de fiebre reumática.

En esta investigación se consideró como variable dependiente la condición de salud bucal, la cual está condicionada por tres aspectos importantes como lo son presencia de caries dental, presencia de enfermedad periodontal, y edentulismo. Como variables explicativas se consideraron variables sociodemográficas, aspectos laborales, personales, económicas y de su entorno. A fin de dar mayor claridad también se tuvo en cuenta como variable explicatoria la posición social que se resolvió a partir de diferentes preguntas que estuvieron en el instrumento adaptado para este estudio de la autora Marcela Arrivillaga^11^.

Los datos para este estudio se obtuvieron durante los meses de enero a marzo del 2020, a partir de la aplicación de un examen clínico realizado en las clínicas odontológicas de la Universidad para el cual se realizó una calibración con un odontólogo que se calibro según la metodología del ENSAB IV, en la cual se evaluaron 20 piezas dentales y se calculó la reproducibilidad inter evaluador obteniendo un valor Kappa De 0,74, igualmente se realizó la calibración con un odontólogo especialista en periodoncia, donde se revisaron 4 pacientes y se calculó la reproducibilidad inter evaluador logrando un valor Kappa de 0,50 para el examen periodontal, igualmente al momento de los exámenes clínicos se contaba con la supervisión del especialista en periodoncia.

Luego de este examen se aplicó un instrumento que contaba con dos apartados para la recolección de la información, el primer apartado tenía que ver con lo relacionado a las variables sociodemográficas y la segunda parte abordaba el contenido de la posición social en el que se tuvo en cuenta que a mayor puntaje mayor posición social (PS) con valores que oscilan entre 7 a 21 puntos; de 7 a 11 puntos representan la posición social baja (PSB), entre 12 y 16 punto la posición social media (PSM) y entre 17 y 21 puntos la posición social alta (PSA) según el autor de referencia.

Se reunieron los registros de las mujeres en una base de datos que se sistematizó en Microsoft Excel por duplicado y mediante la rutina date compare del paquete estadístico Epi Info 3.5.4 se verificó la calidad de la digitación; se efectuaron los correctivos y se exportó la información validada al paquete estadístico STATA 14 para su procesamiento y análisis. Los datos de la investigación están disponibles en el Data- set^12^.

El plan de análisis estadístico univariado incluyó el cálculo de medidas de resumen según la naturaleza y distribución de las variables. Para el análisis bivariado se consideraron como variables de salida la condición bucal, determinada por la presencia o no de caries dental, enfermedad periodontal y edentulismo. Esta variable se relacionó con las variables sociodemográficas, laborales, personales, económicas y de su entorno, al igual que la posición social. Para ello se aplicó test de chi cuadrado o exacto de Fisher para las variables cualitativas y la prueba t de student o test de rangos de Wilcoxon para las cuantitativas dependiendo de la distribución de los datos. A su vez, se consideró un nivel de significancia estadística de alfa <0,05.

Se realizó una prueba piloto con el 10 % de la población, es decir 24 participantes donde surgieron cambios en algunas preguntas de la encuesta, ya que no tenían todas las opciones de respuesta necesarias según las condiciones laborales y personales de las mujeres investigadas.

El presente trabajo se acogió a la normatividad establecida en la Resolución 8430 de 1993, por la que se reglamenta la investigación en salud en Colombia y en la que se preservo la confidencialidad y privacidad de la información aportada mediante el consentimiento informado, el cual fue firmado por los participantes. Adicionalmente, este estudio se sometió a revisión por parte del comité de ética de la Facultad de Odontología de Universidad Santo Tomás seccional Bucaramanga fue avalado mediante acta número 03062017.

## Resultados

La muestra de estudio estuvo conformada por 84 mujeres vinculadas laboralmente a una universidad privada de Santander, Colombia; Según el análisis de las características sociodemográficas, personales, laborales, económicas y de su entorno se evidenció que tienen un promedio de edad de 34,9±11,9, hubo mayor número de participantes que se encontraban en el rango de mayores de 51 años, reportaron no pertenecer a ninguna etnia, nacer en zona urbana y ser solteras (Tabla 1).

Con relación a la labor que desempeña en la universidad, se evidenció que 41,7% eran del sector administrativo con un tipo de vinculación a la Universidad con contrato a término fijo, con jornada laboral de tiempo completo y con un promedio de 9,8±9,6 años vinculadas laboralmente en la Institución (Tabla 1).

En cuanto a su quehacer como madre y mujer se encontró que con relación a si tienen hijos o no la mayoría de las mujeres reportaron tener hijos y en promedio tienen 1,4±1,3, igualmente refirieron que consideran que el tiempo que comparten con sus hijos es poco y en promedio comparten con sus hijos 1,8±1,9 horas al día. Las mujeres participantes manifestaron que en promedio utilizan1,8±1,4 horas diarias para realizar labores del hogar y el 73,8% de ellas dijeron no tener contratada una persona para que les ayuden con las actividades diarias del hogar, con relación al tiempo que dedican a su cuidado personal reportaron dedicarse en promedio 6,4±8,6 horas semanales (Tabla 1). Considerando el factor económico se encontró con mayor frecuencia que las participantes afirmaron no tener otra fuente de ingreso a parte de su salario; En cuanto a la pregunta de si tiene alguna propiedad 46(54,8%) mujeres afirmaron tener alguna propiedad a su nombre.

Con relación a su vivienda y entorno se pudo evidenciar que el 40,5% de las mujeres viven en vivienda alquilada, viven en apartamento y residen en promedio de 3,5±1,5 personas.

**Tabla 1:** Descripción de las variables sociodemográficas, laborales, personales, económicas y de su entorno de las mujeres evaluadas.

Variable	n=84	%
Edad categorizada		
Menos de 20 años	2	2,4
De 21 a 30 años	21	25,0
De 31 a 40 años	20	23,8
De 41 a 50 años	19	22,6
Mayor 51 años	22	26,2
Edad	39,4+11,9 *	39,4+11,9 *
Pertenencia a alguna etnia	15	17,9
¿En qué zona nació?		
Rural	15	17,9
Urbano	69	82,1
Estado Civil		
Casada	33	39,3
Soltera	34	40,5
Divorciada	6	7,1
Viuda	2	2,4
Unión Libre	9	10,7
Rol que desarrolla en la Universidad		
Docente	34	40,5
Administrativo	35	41,7
Servicios Generales	9	10,7
Aprendiz SENA	6	7,1
Tipo de contrato laboral con la Universidad		
Contrato escrito a término Indefinido	5	6,0
Contrato escrito a término fijo	79	94,0
Tipo de Jornada Laboral con la Universidad		
Tiempo completo	76	90,5
Medio tiempo	7	8,3
Catedra	1	1,2
Años de vinculación		
<1 año	15	17,9
2 a 10 años	40	47,6
11 a 20 años	18	21,4
21 a 30 años	6	7,1
>31 años	5	6,0
Variable	n=84	%
Años de vinculación a la Universidad	9,8±9,6*	7(2-13,5) **
Tiene hijos	58	69,0
Considera que el tiempo que comparte con		
sus hijos es:		
Muy poco	20	23,8
Poco	25	29,8
Suficiente	10	11,9
No Aplica	29	34,5
Persona contratada para apoyarlas labores	22	26,2
del hogar		
Número de hijos	1,4±1,3*	1(0-2) **
Número de horas que dedica al día a sus	1,8±1,9*	1(0-3,5) **
hijos		
Número de horas a la semana dedica para su	6,4±8,6*	5(2-7) **
cuidado personal		
Número de horas al día que dedica para	1,8±1,4*	1(1-2) **
realizar labores en su hogar		
Otra fuente de ingreso a parte del salario	35	41,7
Tiene propiedades	46	54,8
Vivienda		
Propia totalmente paga	30	35,7
Propia pagándola	19	22,6
Alquilada	34	40,5
Otra	1	1,2
Tipo de vivienda en que habita la familia		
Casa	37	44,0
Apartamento	47	56,0
*Número de personas que habitan en la* vivienda	3,5±1,5*	3(3-4) **

En la Tabla 2 se puede evidenciar que la prevalencia de caries dental en la población de estudio fue de 85,7%; con relación al edentulismo, la población de mujeres evaluadas registró una prevalencia de 40,5% y en cuanto a la enfermedad periodontal el 79,8% reportaron tener esta patología.

**Tabla 2:** Descripción de las variables de salud bucal.

Variable	n=84	%
Presencia de caries dental	72	85,7
Presencia Edentulismo	34	40,5
Presencia de enfermedad periodontal	67	79,8

### Análisis Bivariado

Las variables sociodemográficas que se asociaron con la presencia de caries dental fueron la edad (p=0,007), etnia (p=0,016), estado civil (p=0,034). Con respecto a la asociación de la presencia de caries dental y las variables laborales se encontró diferencias estadísticamente significativas con las variables años de vinculación cuantitativa (p=0,027) y años de vinculación categorizada (p=0,020).

Al referir las variables personales se observó que, la variable de tiene hijos (p= 0,015) y número de hijos (0,044) se asociaron estadísticamente con la presencia de caries dental. Al analizar si tiene hijos se observa que las mujeres que reportaron tener hijos tenían mayor prevalencia de caries con relación a los que no tienen, y en cuanto al número de hijos la mediana fue de 1(0-2) hijos para las mujeres que presentaron caries mientras que la mediana del número de hijos para las que no tienen caries fue de 2(RIC=1-3).

Al retomar las variables económicas y del entorno se evidenció que ser propietaria de casa o apartamento diferente al que habita (p=0,006) y número de personas que habitan en la vivienda (p=0,048) con la presencia de caries se asociaron de forma significativa.

Al analizar la presencia de enfermedad periodontal relacionada con las variables sociodemográficas, laborales y personales, no se encontraron diferencias estadísticamente significativas; sin embargo, cabe resaltar que la mediana de edad de las mujeres que reportaron tener enfermedad periodontal fue de 39(RIC=29-50), mientras que para las que no padecían enfermedad periodontal fue de 42(RIC=35- 51) años y entre las mujeres que presentaron enfermedad periodontal el 43,3% (n=29) reportaron estar casadas.

Al momento de analizar la presencia de enfermedad periodontal con las variables económicas y de su entorno se hallaron diferencias estadísticamente significativas para las variables: ser propietaria de casa o apartamento donde habita (p=0,026), tener propiedades (p=0,032), su vivienda es (p=0,005), tipo de vivienda en la que habita (p=0,027). En estas variables se evidenció que la mayor frecuencia de las mujeres que presentaron enfermedad periodontal no son propietarias del lugar donde residen.

Con respecto a la presencia de edentulismo y la relación con variables sociodemográficas en la población de estudio, se encontraron diferencias estadísticamente significativas con edad analizada en forma numérica (p=0,000), edad categorizada(p=0,007), zona en que nació (p=0,040) y estado civil (p=0,037). La mediana de edad en las mujeres que registraron edentulismo fue de 46 años comparado con las que no lo registraron que fue de 33 años.

En cuanto a la presencia de edentulismo y las variables laborales, se encontraron asociaciones estadísticamente significativas en las variables, labor en que desarrolla en la universidad (p=0,000), en tipo de jornada laboral tiene con la universidad (p=0,038), años de vinculación en la institución numérica (p=0,000) y categorizada (p=0,000). Con respecto a estas variables el edentulismo fue mayor en las mujeres que desarrollan actividades administrativas, con contrato a término fijo.

Con relación a la presencia de edentulismo y las variables personales, se hallaron las siguientes diferencias estadísticamente significativas: tiene hijos (p=0,009), número de hijos (p=0,000), tiempo que comparte con sus hijos (p=0,005), observándose que la mayoría de las participantes reportaron tener hijos y presentar edentulismo. Teniendo en cuenta las variables económicas y de su entorno con la presencia de la patología mencionada fueron: es propietaria de casa o apartamento donde habita (p=0,044) y tiene otras propiedades (p=0,043). De lo cual se puede resaltar que el 67,6%(n=23) participantes tenían edentulismo y no eran propietarias del lugar donde viven y la mayoría manifestaron no tener propiedades, pero si presentaban edentulismo (Tabla 3).

**Tabla 3:** Relación de variables sociodemográficas y aspectos sociales con condiciones de salud bucal.

	Presencia de caries dental			Presencia de enfermedad periodontal			Presencia de edentulismo		
Variables	Si	No	*Valor-p*	Si	No	*Valor-p*	Si	No	*Valor-p*
	(72)	(12)		(67)	(17)		(34)	(50)	
Edad	36,5(29-	52,5(41-	0,007	39(29-50)	42(35-	0,3818	46(37-	33,5(27-	0,000
	47)	57,5)			51)		54)	43)	
Pertenece a étnica	11(13,9)	3(25,0)	0,016	13(19,5)	2(11,8)	0,913	5(14,7)	10(20,0)	0,461
Zona donde nació			0,113			1,000			0,040
Rural	15(20,8)	---		12(17,9)	3(17,7)		10(29,4)	5(10,0)	
Urbano	57(79,2)	12(100)		55(82,1)	14(82,3)		24(70,6)	45(90,0)	
Estado Civil			0,034			0,476			0,037
Casada	28(38,9)	5(41,7)		29(43,3)	4(23,5)		16(47,1)	17(34,0)	
Soltera	31(43,1)	3(25,0)		25(37,3)	9(52,9)		8(23,5)	26(52,0)	
Divorciada	3(4,2)	3(25,0)		5(7,5)	1(5,9)		4(11,8)	2(4,0)	
Viuda	2(2,8)	---		1(1,5)	1(5,9)		---	2(5,9)	
Unión Libre	8(11,1)	1(8,3)		7(10,4)	2(11,8)		5(10,0)	4(11,8)	
Labor que desarrolla en la Universidad		0,635			0,544			<0,001	
Docente	27(37,5)	7(20,6)		25(37,3)	9(52,9)		10(29,4)	24(48,0)	
Administrativo	31(43,1)	4(33,3)		29(43,3)	6(35,3)		14(41,2)	21(42,0)	
Servicios Generales	8(11,1)	1(8,3)		7(10,4)	2(11,8)		9(26,5)	---	
Aprendiz SENA	6(8,3)	---		6(8,9)	---		1(2,9)	5(10,0)	
Jornada Laboral que tiene			1,000			0,320			0,038
Tiempo completo	65(90,3)	11(91,7)		62(92,5)	14(82,6)		34(100,0)	42(84,0)	
Medio tiempo	6(8,3)	1(8,3)		4(6,0)	3(17,7)		---	7(14,0)	
Catedra	(1(1,2)	---		---	1(1,5)		---	1(2,0)	
Años en la Universidad	6,5(2-12)	14,5(5,5-23)	0,027	7(2-13)	9(5-16)	0,4284*	13(4-20)	5,5(2-9)	0,000
Tiene hijos	46(63,9)	12(100)	0,015	47(70,2)	11(64,7)	0,665	29(85,3)	29(58,0)	0,009
Número de hijos	1(0-2)	2(1-3)	0,044	1(0-2)	1(0-2)	0,3893*	2(1-3)	1(0-2)	0,000
Considera que el tiempo que compar te con sus hijos es:			0,096			1,000			0,005
Muy poco	16(22,2)	4(33,3)		16(23,9)	4(23,5)		14(41,2)	6(12,0)	
Poco	21(29,2)	4(33,3)		20(29,9)	5(29,4)		9(26,5)	16(32,0)	
Suficiente	7(9,7)	3(25,0)		8(11,9)	2(11,8)		5(14,7)	5(10,0)	
No Aplica	28(38,9)	1(8,3)		23(34,3)	6(35,3)		6(17,7)	23(46,0)	
Tiene propiedades	36(50,0)	10(66,7)	0,225	36(53,7)	10(76,5)	0,032	22(58,8)	24(48,0)	0,330
Propietaria de casa o apartamento diferente al que habita	10(12,5)	6(50,0)	0,006	12(17,9)	3(17,7)	1,000	8(23,5)	7(14,0)	0,263
Es propietaria de casa o apartamento donde habita	15(20,8)	3(25,0)	0,500	11(16,4)	7(41,2)	0,026	11(32,4)	7(32,4)	0,044
Tiene otras propiedades	15(20,8)	1(8,3)	0,282	13(19,4)	3(17,7)	1,000	3(8,8)	13(26,0)	0,043
Tipo de propiedad de la vivienda			0,222			0,005			0,281
Propia totalmente paga	23(31,9)	7(58,3)		19(28,3)	11(64,7)		11(32,4)	19(38,0)	
Propia pagándola	16(22,2)	3(25,0)		17(25,4)	2(11,7)		11(32,4)	8(16,0)	
Alquilada	32(44,4)	2(16,67)		31(46,3)	3(17,7)		12(35,3)	22(44,0)	
Otra	1(1,4)	---		---	1(5,9)		---	1(2,0)	
Tipo de vivienda en que la familia			0,222			0,027			0,647
Casa	30(41,7)	7(58,3)		25(37,3)	12(70,6)		16(47,11)	21(42,0)	
Apartamento	42(58,3)	5(41,7)		42(62,7)	5(29,4)		18(52,9)	29(58,0)	
Número de personas que en la vivienda	3,6±1,4	2,7±1,6	0,048	3,4±1,3	3,7±1,9	0,511	3,3+1,6	3,6+1,4	0,413

*Nota: Valor-p Test de Rango de Wilcoxon para variables cuantitativas, Chi Cuadrado de Pearson para variables cualitativas*

Se pudo evidenciar que el 39,3% (n=33) de las mujeres tienen una posición social baja, mientras que el 38,1% (n=32) pertenecen a una posición social media y el 22,6% (n=19) poseen una posición social alta.

Relacionando la presencia de caries con las variables de posición social se evidencia diferencia estadísticamente significativa con la variable que tiene que ver con si la mujer trabajadora de la Universidad es la principal responsable económica de su hogar (p=0,016), donde la mayor frecuencia de las mujeres que reportaron que si eran las responsables principales económicamente de su hogar presentaron caries dental. Es importante señalar que la mayoría de las mujeres que presentaron caries dental se encuentran en una posición social baja.

Al analizar la enfermedad periodontal se evidencia que del total de mujeres que registraron enfermedad periodontal la mayoría se catalogaron en baja posición social y en poca frecuencia en la posición social alta.

Al analizar la presencia de edentulismo con las variables de posición social se evidencian diferencias estadísticamente significativas, con respecto a la variable nivel educativo (p=0,006), ser la principal responsable económica del hogar(p=0,046), cabe resaltar en cuanto a la posición social que 41,2% (n=14) mujeres que presentaron edentulismo se encuentran en una posición social baja siendo la mayoría de la población estudiada, mientras que el 23,5% (n=8) mujeres se encontraban en la posición social alta. (Tabla 4).

**Tabla 4:** Relación de variables de posición social con condiciones de salud bucal.

Variable	Total	Si (72)	No (12)	*Valor-p*	Si (67)	No (17)	*Valor-p*	Si (34)	No (50)	*Valor-p*
Zona de Residencia				0,267			1,000			1,000
Rural	2(2,4)	1(1,4)	1(8,3)		2(3,0)	---		1(2,9)	1(2,0)	
Urbana	82(97,6)	71(98,6)	11(91,7)		65(97,0)	17(100)		33(97,1)	49(98,0)	
Estrato Socioeconómico				0,309			0,433			0,301
Dos	14(16,7)	13(18,1)	1(8,3)		12(17,9)	2(11,8)		8(23,5)	6(12,0)	
Tres	33(39,3)	29(40,3)	4(33,3)		27(40,3)	6(35,3)		15(44,1)	18(36,0)	
Cuatro	23(27,4)	19(26,4)	4(33,3)		19(28,3)	4(23,5)		6(17,7)	17(34,0)	
Cinco	8(9,5)	5(6,9)	3(25,0		6(9,0)	2(11,8)		2(5,9)	6(12,0)	
Seis	6(7,1)	6(8,3)	---		3(4,5)	3(17,6)		3(8,8)	3(6,0)	
Nivel educativo alcanzado				0,414			0,760			0,006
Primaria	1(1,2)	1(1,4)	---		1(1,5)	---		1(2,9)	---	
Secundaria	11(13,1)	10(13,9)	1(8,3)		9(13,4)	2(11,8)		10(29,4)	1(2,0)	
Técnico	20(23,8)	17(23,6)	3(25,0)		16(23,9)	4(23,5)		6(17,7)	14(28,0)	
Tecnología	14(16,7)	14(19,4)	---		13(19,4)	1(5,9)		5(14,7)	9(18,0)	
Universitario	3(3,6)	2(2,8)	1(8,3)		2(3,0)	1(5,9)		1(2,9)	2(4,0)	
Especialización	21(25,0)	16(22,2)	5(41,7)		16(23,9)	5(29,4)		5(14,7)	16(32,0)	
Maestría	14(16,7)	12(16,7)	2(16,7)		10(14,9)	4(23,5)		6(17,7)	8(16,0)	
Régimen de afiliación al sistema de salud				0,857			0,202			1,000
Contributivo	83(98,8)	71(98,6)	12(100)		67(100)	16(94,1)		34(100,0)	49(98,0)	
Especial	1(1,2)	1(1,4)	---		---	1(5,9)		---	1(2,0)	
Trabajadora exclusivamente de la Institución donde se realizó el estudio				0,167			0,762			0,336
Si	62(73,8)	55(76,4)	7(58,3)		50(74,6)	12(70,6)		27(79,4)	35(70,0)	
Nivel de ingreso mensual (SM- MLV)				0,165			0,742			0,710
1 salario (877,803)	38(45,2)	35(48,6)	3(25,0)		32(47,8)	6(35,3)		17(50,0)	21(42,0)	
2 salarios (1,755,606)	9(10,7)	7(9,7)	2(16,7)		7(10,5)	2(11,8)		5(14,7)	4(8,0)	
3 a 4 salarios (2,633,490/ 3,511,320)	15(17,9)	14(19,4)	1(8,3)		12(17,9)	3(17,7)		5(14,7)	10(20,0)	
5 a 6 salarios (4,389,150/ 5,266,890)	19(22,6)	14(19,4)	6(41,7)		14(20,8)	5(29,4)		6(17,7)	13(26,0)	
Propiedades a su nombre				0,342			0,117			0,422
Si, 1 propiedad	32(38,1)	26(36,1)	6(50,0)		22(32,8)	10(58,8)		16(47,1)	16(32,0)	
Si, más de una propiedad	14(16,7)	11(15,3)	3(25,0)		11(16,4)	3(17,7)		5(14,7)	9(18,0)	
No	38(45,2)	35(48,6)	3(25,0)		34(50,8)	4(23,5)		13(38,2)	25(50,0)	
Crédito con entidades financieras o bancos a su nombre				0,458			0,401			0,876
No	15(17,9)	14(19,4)	1(8,3)		14(20,9)	1(5,9)		5(14,7)	10(20,0)	
Si, por montos o suma hasta 20 millones de pesos	38(45,2)	33(45,8)	5(41,7)		29(43,3)	9(52,)		16(47,1)	22(44,0)	
Si, Por montos o sumas superiores a 20 millones	31(36,9)	25(34,7)	6(50,0)		24(35,8)	7(41,2)		13(38,2)	18(36,0)	
Posición social categórica				0,246			0,374			0,909
Baja	33(39,3)	30(41,7)	3(25,0)		28(41,8)	5(29,4)		14(41,2)	19(38,0)	
Media	32(38,1)	28(38,9)	4(33,3)		26(38,8)	6(35,3)		12(35,3)	20(40,0)	
Alta	19(22,6)	14(19,4)	5(41,7)		13(19,4)	6(35,3)		8(23,5)	11(22,0)	

*Nota: Valor-p Chi Cuadrado de Pearson y Exacta de Fisher*

## Discusión

Las patologías bucales, como la caries dental, la enfermedad periodontal y el edentulismo son más comunes de lo que se piensa, pues afectan a más de la mitad de la población mundial; además estas condiciones tienen una alta prevalencia si se comparan con otras alteraciones que se presentan a nivel de la cavidad bucal del ser humano^13^;Sin embargo, hay que revisar que el estado de salud general y en especial el de la boca está condicionado por diferentes determinantes que influyen en la salud de las personas, tales como el género, el nivel educativo, ingresos socioeconómicos, clase social, posición social entre otros.

En relación con la problemática expuesta, los resultados del presente estudio mostraron que las participantes tenían una mayor prevalencia de caries dental con un 85,7%, seguido de la enfermedad periodontal con un 79,8%, y menor prevalencia de edentulismo con un 40,5%; al compararse estos datos con los resultados del ENSAB IV se puede evidenciar que se obtuvieron datos no coincidentes, ya que la caries dental y la enfermedad periodontal en las mujeres de edades entre 20 a 34 años fue de 54,29% y 18,49% y 33,22% y las mujeres de 35 a 44 años fue de 60,97%, mujeres de 45 a 64 años fue de 54,79% y 53,97% respectivamente, lo cual evidencia que son dos condiciones bucales que existen con una alta frecuencia en las mujeres colombianas, pero sobre todo se dieron con una mayor presencia en las mujeres que laboran en la universidad, lo que se pudo dar por los diferentes aspectos sociales que influyen en la aparición de dichas patologías^14^.

En este sentido al comparar esta investigación con resultados del estudio de Ahumada 2017 en Chile, el cual reportó que las mujeres tenían una prevalencia de caries dental de 99,6%, al igual que Olmos 2013 en Uruguay donde reportaron una prevalencia de 94%, lo cual evidencia una alta prevalencia de esta patología en países suramericanos. Sin embargo, los resultados obtenidos difieren con el estudio de Kamberi y col. en la República de Kanovo al Sureste de Europa en el 2016, ya que reportaron que las mujeres tenían una prevalencia de caries de 57,1%, lo que connota una menor prevalencia entre los países analizados con mayor desarrollo económico^15^^-^^17^.

Con referencia a los resultados obtenidos de la presencia de enfermedad periodontal se puede evidenciar una prevalencia de 79,8%, lo que difiere un poco con los resultados obtenidos por Fuentes en el 2015 en donde la prevalencia de enfermedad periodontal considerada como gingivitis fue de 98,3 % para las mujeres, establecida mediante el índice gingival de Loe & Sillness; mientras que la prevalencia de enfermedad periodontal, medida a partir del examen periodontal básico, el cual es una variación del CPTIN determinó que los códigos EPB3(sondaje entre 4-6 mm) y EPB4(sondaje 6 o más) tenían una prevalencia de 22,9%, lo que indica que fueron los códigos más frecuentes en la mayor cantidad de sextantes evaluados en las mujeres sujeto de estudio lo cual es un resultado muy diferente a este trabajo ya que se evidenció que el código que se presentó con mayor frecuencia fue el tres, es decir presencia de cálculo dental^18^.

Para el edentulismo se observó que las participantes presentaban una prevalencia de 40,5%, lo cual difiere con lo reportado por Cortés y col en el 2013 en el estudio realizado a población Chilena debido a que del total de evaluados un 89,7% registró ausencia de al menos de uno de sus dientes; según Avendaño en el 2016 en su investigación sobre prevalencia de edentulismo evidenció que el 63,6% de la población femenina estudiada presentaba algún tipo de edentulismo, igualmente lo estipularon Escudero y colaboradores en el 2020 en donde estudiaron la prevalencia de edentulismo con relación a la calidad de vida y los resultados fueron que el 59% de la población femenina encuestada presentaba edentulismo, lo cual es similar al presente estudio, ya que evidencian que las mujeres son las que muestran mayor presencia de edentulismo con relación a los hombres, debido a los cambios hormonales que enfrentan las mujeres en sus diferentes ciclos y etapas de la vida, los cuales afectan los tejidos periodontales hasta llegar a la pérdida de las estructuras dentarias y en esta investigación se pueden evidenciar una variabilidad en los ciclos de vida las participantes lo que puede dar respuesta a este evento^19^^-^^21^.

En lo referente a la relación de la salud bucal, considerando las tres patologías estudiadas con los factores sociodemográficos y sociales que se tuvieron en cuenta en las mujeres trabajadoras se pueden evidenciar las siguientes relaciones que determinan la influencia de estos factores en la salud bucal de los sujetos participantes.

Al momento de estudiar la relación de presencia de caries dental con la edad de los participantes en la presente investigación se evidenció una diferencia estadísticamente significativa, puesto que la mediana de la edad para las mujeres que tenían caries dental era de 36,5(29-47), mientras que las que no era de 52,5(41-57,5), lo cual es opuesto con el estudio de Mafla en 2019, los cuales mostraron que en las mujeres la edad es un factor de riesgo que incrementa el índice de caries, siendo el grupo de 45-54 años el más alto, es decir que para ellos entre mayor edad existe más probabilidad de tener esta condición. Sin embargo, se encontró algo similar con los resultados de esta investigación y lo expresado por Kamberi y col. en el 2016, quienes manifestaban que los participantes con mayor presencia de caries dental tenían entre 18 y 34 años (44,2%), seguidos de los de 35 a 44 años (18,3%), entretanto que los que tenían más bajo porcentaje eran los participantes de 75 años o más (2,9%), lo cual se puede explicar por qué las mujeres con mayor edad están en la etapa de la menopausia y tienden a tener más problemas periodontales, lo que a su vez genera pérdida de dientes 22 y puede conllevar a tener menos presencia de caries dental y en el caso de este estudio la mayoría de población tenía 51 años o más^17^^,^^23^.

Con respecto a la relación de la enfermedad periodontal, cabe resaltar que todos los grupos de edad tuvieron representación es decir, que en las pacientes estudiadas al menos una presentó enfermedad periodontal y la mediana de edad para las mujeres que tenían esta patología fue de 39(29-50) estos resultados difieren con Fuentes en el 2015 en el cual en su trabajo de investigación en Chile obtuvo como resultado que las personas con mayor prevalencia de enfermedad periodontal considerándose como gingivitis fueron los participantes con edad mayor a 50 años y de género femenino17. Según Martelli en 2017 en su estudio realizado demostró que la periodontitis afecta más a las mujeres que a los hombres y se presenta con mayor frecuencia en mujeres menores de 35 años lo cual difiere de lo reportado en el presente estudio; No obstante, un estudio realizado en población cubana se evidenció que la población más afectada por la enfermedad periodontal considerada como gingivitis fue la población joven , mientras que para la población adulta predominó la periodontitis en las mujeres estudiadas; lo que conlleva a pensar que la enfermedad periodontal para las mujeres es una patología que se presenta con un alta frecuencia en los diferentes ciclos de vida acompañada por el cambio hormonal que se presenta en las diferentes etapas de la vida como mujer^24^^-^^25^.

Con respecto a la asociación encontrada entre edentulismo y edad, es importante mencionar que se evidenciaron diferencias estadísticamente significativas en mujeres cuya particularidad presentan un rango de edad entre 37 y 54 años siendo más frecuente en las mayores de 50 años situación similar a la referida por Luengas- Aguirre y col en 2015 quienes evidenciaron mayor frecuencia de edentulismo en la población femenina adulta mayor. Sin embargo, lo anterior parece no coincidir con los resultados presentados por Escudero y col en 2020 quienes reportaron mayor frecuencia de edentulismo parcial en el grupo de mujeres entre los 15 y 24 años^21^^,^^26^. Lo anterior, permite deducir que la pérdida de estructuras dentarias se da como consecuencia de patologías orales como caries dental y enfermedad periodontal y que estas a su vez están determinadas por diversos factores de tipo social^27^.

Con relación al nivel educativo de las participantes y su relación con su estado de salud bucal se observó que gran parte de la población estudiada padecían de las patologías bucales estudiadas, sin embargo, hay que tener en cuenta que las personas que más tienen caries dental y enfermedad periodontal fueron las mujeres que tenían estudios técnicos y las que más padecían de edentulismo fueron las de niveles educativos inferiores como secundaria, lo cual es similar con el estudio de Arrica y colaboradores en el 2017 en donde su investigación encontraron que las personas con un bajo nivel educativo tenían un peor estado de salud bucal^28^.

Igualmente, un estudio realizado en tres países de América Latina reportó que tener más años de estudio se asocia positivamente con tener mejor estado de salud. Lo anterior lleva a tener en cuenta que el nivel educativo es un aspecto relacionado con la posición social influyente en la salud bucal de las personas y en este caso de las mujeres^29^.

En cuanto a la labor que desarrollaban las mujeres en la universidad relacionado con la posición social y con las patologías bucales es importante señalar que las mujeres que tienen actividades laborales con menos desempeño académico tienen mayor presencia de caries dental. Lo anterior permite inferir que las mujeres con cargos inferiores ya sea por nivel educativo o nivel de ingresos tienen mayor posibilidad de padecer estas patologías, lo cual es similar con la investigación de Morita y col, realizada en Japón a hombres trabajadores, donde se encontró que las personas que eran profesionales es decir con mayor estudio tenían mayor número de dientes sanos y naturales, mientras que los sujetos que asumían laborales como los conductores y ocupaciones de servicio poseían mayor número de dientes enfermos y dientes faltantes; lo cual conlleva a inferir que la relación de labor o el trabajo está relacionado con el estado de salud oral o salud en general que tienen las personas^30^.

Así mismo, al analizar el estrato socioeconómico asociado a la presencia de las diferentes patologías estudiadas se encontró que las mujeres pertenecientes al estrato socioeconómico tres eran las que más presentaban caries dental, enfermedad periodontal y edentulismo, comparado con las mujeres de estratos más altos como el cinco y el seis. Lo anterior es similar a lo presentado en un artículo desarrollado en México en donde evidenciaron que las personas que se encuentran en niveles socioeconómicos bajos tienden a presentar desigualdades que demarcan la severidad de la caries dental y enfermedad periodontal, así mismo son indicadores de la alta frecuencia de perdida dental en el grupo de personas menos favorecidas^26^.

Según Peñuela Munévar expresa que “la desigualdad entre los estratos socioeconómicos sugiere inequidades y dificultades para acceder a la atención en servicios odontológicos y hay una sugerente brecha en el número de dientes cariados y obturados en el estrato uno comparado con el cinco”, esta idea es relevante y similar a lo encontrado en la presenta investigación, lo anterior soporta que el nivel o estrato socioeconómico es uno de los factores sociales más influyentes en la salud bucal^31^.

En cuanto a la relación de las patologías caries dental y edentulismo con la pregunta a las mujeres de si son o no las principales responsables económicamente del hogar se pudo concretar que existe una diferencia estadísticamente significativa en cuanto a esta relación, debido a que las mujeres con mayor presencia de caries dental y edentulismo son las responsables de la parte económica del hogar, lo cual es similar con los resultados reportados por el ENSAB IV donde se reportó que las personas que se encuentran en las fracciones sociales capa media pudiente y capa media pobre, en las cuales se puede ubicar a la población de este estudio presentan una mayor prevalencia de caries dental, enfermedad periodontal y edentulismo^14^.

Al analizar la posición social como variable para determinar el gradiente social relacionado con la caries dental, la enfermedad periodontal y el edentulismo, en el presente estudio no se evidenciaron diferencias estadísticamente significativas: sin embargo, es importante dar a conocer la correlación de estas condiciones en la población de estudio.

Por lo anterior, se evidenció que la mayoría de las mujeres que tenían estas patologías en su boca se encontraban en una posición social baja, mientras que las que estaban en una posición social alta tenían menos presencia de estas enfermedades en su boca; esto es similar a lo reportado por la Organización Mundial de la Salud Regional de Europa donde mencionan que normalmente hay un deterioro continuo de la salud, lo cual evidencia visiblemente que la mortalidad y morbilidad aumenta a medida que aumenta el grado de desventaja social^32^.

Así mismo, existe congruencia con autores como Beebe-Dimmer y col en 2004, quienes reportaron que, durante las últimas décadas, ha aumentado la brecha relativa en las tasas de mortalidad entre los grupos socioeconómicos altos y bajos en varios países europeos. Sabbah y col en el 2007 evidenciaron mayor prevalencia de una mala percepción de salud bucal y general, enfermedad periodontal y cardiopatía isquémica en los niveles socioeconómicos más bajos, relación que registran mayores índices de pobreza, menores ingresos y nivel de educación. Neil y col en el 2020 dieron a conocer, a partir de una revisión de literatura, que la posición socioeconómica de las mujeres influye de manera constante en su salud a lo largo del curso de la vida, ya que en varias culturas las mujeres tienen bajo acceso a los recursos socioeconómicos que garantizan la buena salud y el acceso a la atención^33^^-^^35^.

La presente investigación evidencia fortalezas relacionadas con el tipo de población seleccionada para el estudio y la relación de esta con el gradiente social, ya que en la literatura no se evidencian artículos de esta temática desarrollados solo con mujeres como objetivo de estudio, igualmente fue significante estudiar las diferentes características que se evaluaron de cada participante para determinar las condiciones personales, laborales, económicas y de su entorno, para así general un perfil más claro de las circunstancias que tienen las mujeres que influyen en su salud bucal.

Con relación a las limitaciones de este estudio cabe mencionar que por las condiciones de excepción derivadas de la pandemia del Covid-19, no fue fácil vincular a las participantes al proyecto, por tal motivo se hizo necesario desarrollar este trabajo en la población de estudio más cercana al área de trabajo, que contaran con disponibilidad en el tiempo de la investigación para el examen odontológico; por lo tanto la población fue seleccionada por conveniencia originando un potencial sesgo de selección y de esta manera menor poder de generalización de los resultados a la población de mujeres de la universidad.

## Conclusiones

El gradiente social evaluado como posición social no tuvo diferencias estadísticamente significativas, sin embargo, si se estableció que las mujeres que se ubicaron según los factores sociales estudiados en la posición social alta tenían menos carga de enfermedades bucales; mientras que las mujeres que se encontraban en la posición social baja tenían mayor prevalencia de caries dental, enfermedad periodontal y edentulismo, las cuales evidencian el estado de salud de la población de estudio; igualmente cabe señalar que los factores sociales influyentes en el estado de salud bucal de las participantes fueron edad, estado civil, nivel educativo, labor que realiza diariamente, estrato socioeconómico, responsabilidad económica dentro del hogar.
